# Paracentesis Thoracis

**Published:** 1874-06

**Authors:** S. B. Ward


Paracentesis Thoracis—*By S. B. Ward, M.D.*—It is well known that when any considerable amount of fluid is present in the peritoneal cavity, or when an ovarian cyst is filled with fluid, by tapping one side of the cavity, no matter how slightly, a wave is started, which is transmitted in all directions, the shock produced by which can be readily felt at any point of the cavity. So far as I know, it has never been pointed out that the same wave-shock can be transmitted from the top to the bottom of the chest filled with fluid; or, in case the chest is only partly filled, from any point below the level of the fluid to any other point below the same level.

